# Supplementary material for: The Validity and Reliability of the PHQ-9 and PHQ-2 on Screening for Major Depression in Spanish Speaking Immigrants in Chile: A Cross-Sectional Study
Source: Int J Environ Res Public Health. 2022 Oct 27;19(21):13975. doi: 10.3390/ijerph192113975 (PMC9655214; doi:10.3390/ijerph192113975)
Supplement: Supplementary file 1 [file ijerph-19-13975-s001.zip › ijerph-1924715-supplementary.pdf]

Supplementary Tables and Figure

Figure S1. Tested models of the Spanish version of PHQ-9
Table S1. Distribution of PHQ scores and 12 month-CIDI Major Depressive Disorder by immigrant group
Table S2. Internal consistency of the Chilean version of the PHQ-9 and PHQ-2 (n=897)
Table S3. Internal consistency of the Chilean version of the PHQ-9 by immigrant group
Table S4. Internal consistency of the Chilean version of the PHQ-2 by immigrant group
Table S5. Goodness of fit indices for PHQ-9 factor models by immigrant group
Table S6. Convergent validity coefficients of the PHQ-9 and PHQ-2 by immigrant group

Figure S1. Tested models of the Spanish version of PHQ-9

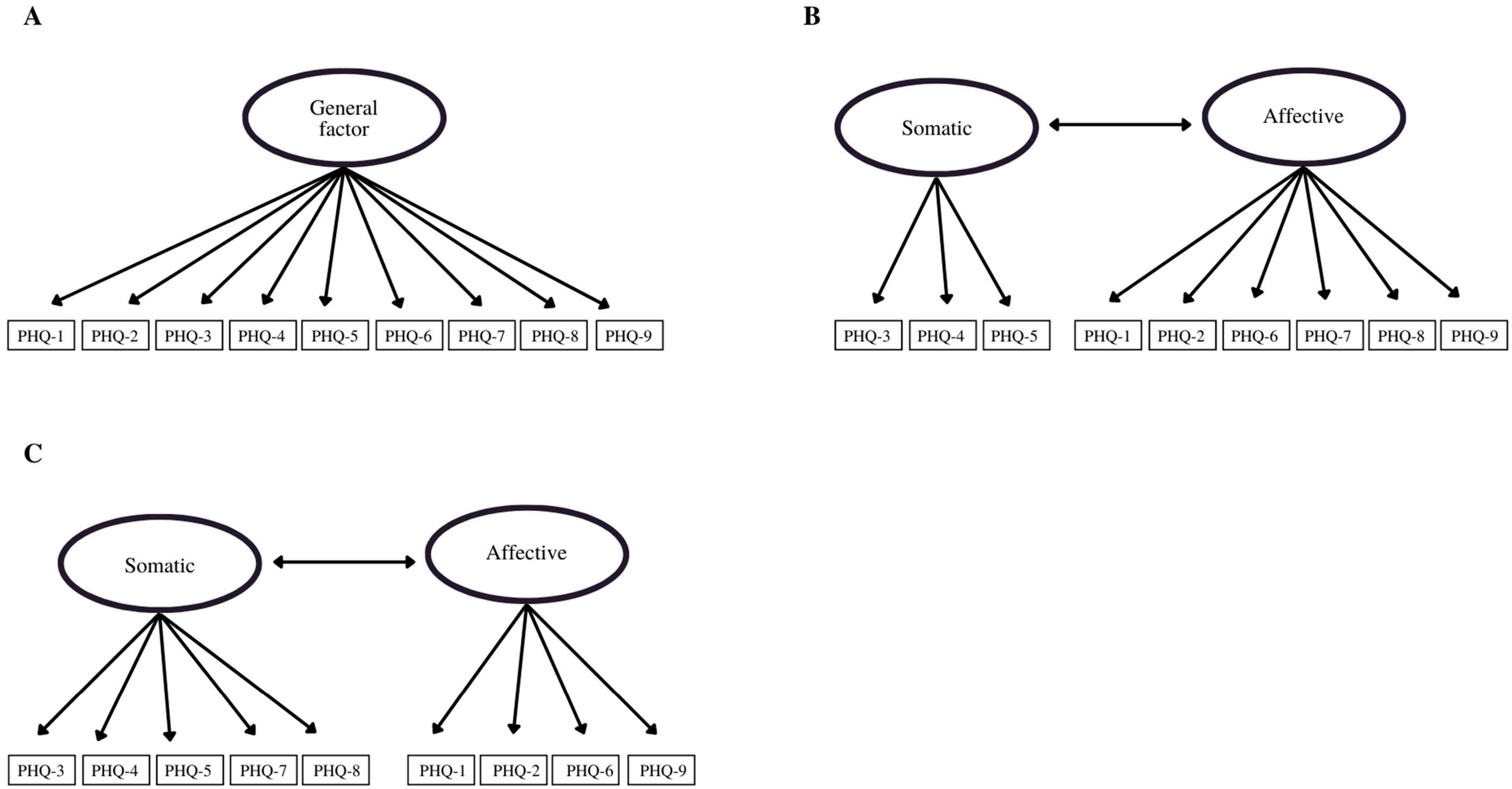

Note: A: Originally validated one-factor model of Kroenke [1] (Model 1); B: Two-factor model of Krause [2] (Model 2a); C: Two-factor model of Richardson and Richard [3] (Model 2b).

Table S1. Distribution of PHQ scores and 12 month-CIDI Major Depressive Disorder by immigrant group

	Colombia (n=94)				Peru (n=333)				Venezuela (n=375)				Other LA (n=95)			
Questionnaire			CIDI				CIDI				CIDI				CIDI	
Symptom severity	n	(%)	negative	positive	n	(%)	negative	positive	n	(%)	negative	positive	n	(%)	negative	positive
PHQ-9																
None (0-4)	85	(89.0)	85	0	286	(83.0)	286	0	335	(88.0)	334	1	84	(87.9)	84	0
Mild (5-9)	7	(8.3)	6	1	27	(9.8)	26	1	34	(10.3)	32	2	5	(5.6)	5	0
Moderate (10-14)	1	(1.9)	1	0	11	(4.2)	11	0	3	(1.0)	3	0	4	(2.3)	4	0
Severe (≥15)	1	(0.8)	1	0	9	(2.9)	8	1	3	(0.7)	3	0	2	(4.2)	2	0
PHQ-2																
Minimum (0-2)	89	(91.9)	88	1	312	(92.8)	312	0	363	(96.3)	360	3	91	(98.6)	91	0
Mild (≥3)	5	(8.1)	5	0	21	(7.2)	19	2	12	(3.7)	12	0	4	(1.4)	4	0
Total	94	(100.0)	93	1	333	(100.0)	331	2	375	(100.0)		3	95	(100.0)	95	0

Note: Number of cases unweighted and % weighted.

Composite International Diagnostic Interview (CIDI); Latin America (LA); 9-item Patient Health Questionnaire (PHQ-9); 2-item Patient Health Questionnaire (PHQ-9).

Table S2. Internal consistency of the Chilean version of the PHQ-9 and PHQ-2 (n=897)

	all (n=897)			
Version<br>Item	Corrected item-total correlation	Alpha if item deleted	Overall Cronbach's alpha	Spearman-Brown
PHQ-9				
1. Anhedonia	0.610	0.899	0.904	
2. Depressed mood	0.724	0.890		
3. Sleep problems	0.687	0.893		
4. Low energy	0.760	0.887		
5. Appetite changes	0.669	0.894		
6. Low self-esteem	0.706	0.891		
7. Concentration difficulty	0.689	0.893		
8. Psychomotor agitation or retardation	0.655	0.895		
9. Suicidal ideation	0.682	0.897		
PHQ-2				
1. Anhedonia	0.604		0.746	0.620***
2. Depressed mood	0.604			

Note: 9-item Patient Health Questionnaire (PHQ-9); 2-item Patient Health Questionnaire (PHQ-9).
*** $p \leq 0.001$

Table S3. Internal consistency of the Chilean version of the PHQ-9 by immigrant group

	Colombia (n=94)			Peru (n=333)			Venezuela (n=375)			Other LA (n=95)		
Item	Corrected item-total correlation	Alpha if item deleted	Overall Cronbach's alpha	Corrected item-total correlation	Alpha if item deleted	Overall Cronbach's alpha	Corrected item-total correlation	Alpha if item deleted	Overall Cronbach's alpha	Corrected item-total correlation	Alpha if item deleted	Overall Cronbach's alpha
1. Anhedonia	0.451	0.900	0.887	0.795	0.939	0.946	0.524	0.838	0.847	0.578	0.881	0.886
2. Depressed mood	0.540	0.887		0.845	0.936		0.634	0.827		0.747	0.870	
3. Sleep problems	0.845	0.859		0.783	0.940		0.630	0.826		0.609	0.877	
4. Low energy	0.735	0.866		0.810	0.939		0.653	0.823		0.829	0.856	
5. Appetite changes	0.782	0.861		0.741	0.943		0.566	0.832		0.599	0.877	
6. Low self-esteem	0.683	0.872		0.812	0.939		0.595	0.829		0.709	0.868	
7. Concentration difficulty	0.622	0.876		0.812	0.939		0.550	0.834		0.672	0.871	
8. Psychomotor agitation or retardation	0.712	0.875		0.842	0.937		0.575	0.832		0.631	0.881	
9. Suicidal ideation	0.738	0.873		0.723	0.943		0.555	0.841		0.700	0.880	

Note: Latin America (LA).
* p≤ 0.05; ** p≤ 0.01 *** p≤ 0.001

Table S4. Internal consistency of the Chilean version of the PHQ-2 by immigrant group

	Colombia (n=94)			Peru (n=333)			Venezuela (n=375)			Other LA (n=95)		
Item	Corrected item-total correlation	Overall Cronbach's alpha	Spearman - Brown	Corrected item-total correlation	Overall Cronbach's alpha	Spearman -Brown	Corrected item-total correlation	Overall Cronbach's alpha	Spearman -Brown	Corrected item-total correlation	Overall Cronbach's alpha	Spearman -Brown
1. Anhedonia	0.398	0.567	0.503***	0.797	0.884	0.725***	0.450	0.582	0.465***	0.624	0.766	0.731***
2. Depressed mood	0.398			0.797			0.450			0.624		

Note: Latin America (LA).
* p≤ 0.05; ** p≤ 0.01 *** p≤ 0.001

Table S5. Goodness of fit indices for PHQ-9 factor models by immigrant group

Model	Fit values				Factor correlation
Immigrant group	χ^2 $_{(df)}$	CFI	RMSEA (90% CI)	SRMR	
One factor 1					
Colombia	$188.508_{(27)}$***	0.764	0.144*** (0.040-0.247)	0.078	
Peru	$299.707_{(27)}$***	0.931	0.135*** (0.089-0.182)	0.047	
Venezuela	$309.755_{(27)}$***	0.884	0.107*** (0.039-0.164)	0.074	
Other LA	$184.314_{(27)}$***	0.599	0.175*** (0.086-0.257)	0.095	
All (n=897)	$515.414_{(27)}$***	0.915	0.117*** (0.087-0.149)	0.052	
Two factor 2a					
Colombia	$135.412_{(26)}$***	0.881	0.104*** (0.010-0.207)	0.077	
Peru	$249.898_{(26)}$***	0.950	0.117*** (0.066-0.166)	0.043	
Venezuela	$240.663_{(26)}$***	0.946	0.073*** (0.015-0.139)	0.069	
Other LA	$178.692_{(26)}$***	0.596	0.179*** (0.092-0.261)	0.102	
All (n=897)	$405.081_{(26)}$***	0.942	0.099*** (0.066-0.132)	0.050	0.87***
Two factor 2b					
Colombia	$188.237_{(26)}$***	0.748	0.152*** (0.044-0.255)	0.078	
Peru	$285.922_{(26)}$***	0.935	0.133*** (0.085-0.181)	0.046	
Venezuela	$303.827_{(26)}$***	0.886	0.109*** (0.038-0.167)	0.075	
Other LA	$181.352_{(26)}$***	0.597	0.179*** (0.089-0.262)	0.113	
All (n=897)	$508.634_{(26)}$***	0.917	0.119*** (0.087-0.151)	0.052	0.97***

Note: Robust Comparative Fit Index (CFI); confidence interval (CI); degrees of freedom (*df*); Latin American (LA); Robust Root Mean Square Error of Approximation (RMSEA); Standardized Root Mean Square Residual (SRMR); Chi-square (χ^2).

*** $p < 0.001$

Model 1: Originally validated one-factor model of Kroenke [1].

Model 2a: Two-factor model of Krause [2] with item 3, 4 and 5 loaded on one somatic factor and the six remaining items loaded on an affective factor .

Model 2b: Two-factor model of Richardson and Richard [3] with item 3,4,5,7,8 loaded on the somatic factor and four remaining items loaded on an affective factor.

Table S6. Convergent validity coefficients of the PHQ-9 and PHQ-2 by immigrant group

	Colombia (n=94)		Peru (n=333)		Venezuela (n=375)		Other LA (n=95)		all (n=897)	
	PHQ-9	PHQ-2	PHQ-9	PHQ-2	PHQ-9	PHQ-2	PHQ-9	PHQ-2	PHQ-9	PHQ-2
PHQ-9	-	0.689***	-	0.828***	-	0.729***	-	0.566***	-	0.714***
PHQ-2	0.689***	-	0.828***	-	0.729***	-	0.566***	-	0.714***	-
GAD-7	0.607***	0.442***	0.643***	0.581***	0.617***	0.459***	0.595***	0.532***	0.625***	0.520***

Note: General Anxiety Disorder 7 items (GAD-7); Latin American (LA); 9-item Patient Health Questionnaire (PHQ-9); 2-item Patient Health Questionnaire (PHQ-9).

*** p≤ 0.001

References

1. Kroenke K, Spitzer RL, Williams JB: The PHQ-9: validity of a brief depression severity measure. *J Gen Intern Med* 2001, 16(9):606-613.
2. Krause JS, Bombardier C, Carter RE: Assessment of depressive symptoms during inpatient rehabilitation for spinal cord injury: is there an underlying somatic factor when using the PHQ? *Rehabilitation Psychology* 2008, 53(4):513.
3. Richardson EJ, Richards JS: Factor structure of the PHQ-9 screen for depression across time since injury among persons with spinal cord injury. *Rehabilitation Psychology* 2008, 53(2):243-249.
